# On the manipulator-focused response to manipulation cases

**DOI:** 10.1007/s11098-024-02218-3

**Published:** 2024-10-14

**Authors:** Gabriel De Marco, Taylor W. Cyr

**Affiliations:** 1 Faculty of Philosophy, Oxford Uehiro Centre for Practical Ethics, University of Oxford, Oxford, UK; 2 Department of Classics and Philosophy, Samford University, Birmingham, USA

**Keywords:** Moral responsiblity, Free action, Manipulation argument, Soft-line replies, Manipulator-focused

## Abstract

In this paper, we identify a class of responses to cases of manipulation that we label *manipulator-focused views*. The key insight of such views is that being subject to the will of another agent significantly affects our freedom and moral responsibility. Though different authors take this key insight in different directions, and the mechanics of their views are quite different, these views turn out to share many key components, and this allows us to discuss several authors’ views at the same time, highlighting a variety of challenges for such views and helping to identify pitfalls to avoid in further developments of views of this type. Moreover, as we survey manipulator-focused views and the challenges that plague them, we go beyond the typical problem cases for such views—natural force variations of manipulation cases—and introduce several new manipulation cases. We conclude by comparing the prospects for this family of views with its main rival, namely bypassing views.

## Introduction

### Chuck and Sally

Consider a pair of cases from Alfred Mele:

*Chuck* Chuck enjoys killing people…When he was much younger, Chuck enjoyed torturing animals, but he was not wholeheartedly behind this….He freely set out to ensure that he would be wholeheartedly behind his torturing of animals and related activities, including his merciless bullying of vulnerable people, and he was morally responsible for so doing…His strategy worked. Today, he stalked and killed a homeless man, Don. (2019: 19–20)

*Sweet Sally* When [Sweet] Sally crawled into bed last night, she was one of the kindest, gentlest people on Earth…Sally’s character was such that intentionally doing anyone serious bodily harm definitely was not an option for her: Her character—or collection of values—left no place for a desire to do such a thing to take root…But Sally awakes with a desire to stalk and kill a neighbor, George. Although she had always found George unpleasant, she is very surprised by this desire. What happened is that, while Sally slept, a team of psychologists that had discovered the system of values that make Chuck tick implanted those values in Sally after erasing her competing values. They did this while leaving her memory intact, which helps account for her surprise. Sally reflects on her new desire. Among other things, she judges, rightly, that it is utterly in line with her system of values…Seeing nothing that she regards as a good reason to refrain from stalking and killing George, provided that she can get away with it, Sally devises a plan for killing him; and she executes it—and him—that afternoon… (2019: 20–21)

Although Sweet Sally and Chuck have similar motivations and values relevant to the decision at the time of their respective killings, and although they may have similar abilities or capacities to recognize and respond to reasons, or to deliberate on the basis of informed deliberation, many will judge that, while Chuck is morally responsible for killing Don, Sally is not morally responsible for murdering George.^1^

Given the similarities between Chuck and Sally at, and just before, the time of their killings, various philosophers have argued that an agent’s moral responsibility depends on more than simply facts about features internal to the agent at, or shortly before, the time of action.^2^ There are, roughly, two main families of views that incorporate this insight, which we will call *bypassing views*, and *manipulator-focused views*. Our goal in this paper is to evaluate the latter, and we do this by focusing on particular versions of manipulator-focused views. But first, we introduce both types of views and the current dialectic in more detail.

### Bypassing views

On *bypassing views*, the relevant difference between agents like Chuck and Sally is, in part, that unlike Chuck, the attitudes leading to Sally’s decision were acquired or modified in a way that bypassed her capacities for control over her mental life (De Marco 2023a; Fischer, 2012: Chapter 11; Haji & Cuypers, 2008; McKenna, 2016; Mele, 1995, 2006, 2019). The relevant capacities are, for instance, the capacity to critically assess, endorse, and sustain one’s values (Fischer, 2012: 198; Haji & Cuypers, 2008: 30; McKenna, 2016: 97; Mele, 1995: 118–20).

Bypassing theorists are not committed to the claim that an action’s issuing from an attitude that was acquired via bypassing is sufficient to eliminate an agent’s responsibility for that action.^3^ As many have pointed out, we are subject to a variety of influences in our daily lives, and it is quite possible that many of these influence our attitudes via bypassing.^4^ Consider, for example, Mele’s *mild manipulation* case. Carl has made a commitment to refrain from eating snacks for six months, yet daily experiences a few medium-strength desires to eat a snack. Although the urge is always resistible, he occasionally acts on it. Suppose now that a manipulator induces in Carl such an urge about once a day, at times during which Carl would not acquire these urges in the normal way, and Carl succumbs to it about 5% of the time. As Mele suggests, whether Carl is morally responsible for eating snacks in response to these urges “is implausibly regarded as turning on whether the urges are produced, on the one hand, in the ‘normal’ way or…, on the other, by a manipulator who flashes subliminal ‘snack’ messages at him” (2019: 37). In order to avoid these problems, bypassing theorists offer more nuanced accounts. We return below to cases of mild manipulation.

Importantly, bypassing views make no reference to the presence of a manipulator in explaining why agents like Sally lack responsibility for their deeds, since bypassing can occur as a result of natural forces. Consider a *natural force* case:

*Natalie* Natalie was just as sweet as Sally was before her transformation. However, due to a strange electro-magnetic storm over her house while Natalie slept last night, she underwent a change much like Sally’s. She wakes up, deliberates in a similar fashion, decides to kill her neighbor, and does so.^5^

Many will think that Natalie, like Sally, is not responsible for killing her neighbor, and bypassing views can offer the same explanation for her lack of responsibility.

Bypassing views are designed to account for cases like these, in which an adult agent undergoes a radical reversal—either as a result of manipulation, or of natural forces—and is intuitively not responsible for some action or other. We call such cases *mid-life manipulation* cases.

### Original design and manipulator-focused views

Another group of cases, which we call *original design* cases, pose a problem for bypassing views. Consider the following case introduced by Mele (2006: 188–9):

*Ernie* Diana, a goddess with special powers and knowledge, wants event E to occur 30 years after time t. Diana knows what the state of the universe is just before t and she knows the laws of nature of her deterministic universe. With these things in mind, she creates a zygote, Z, in Mary at time t which will develop into Ernie. Diana does this knowing that, given the state of the world and the laws, Ernie will, 30 years in the future, perform action A which will bring about event E.

Compare Ernie to Bernie, an agent exactly like Ernie but for the fact that his zygote came about in the ordinary way in his deterministic world. Like the case of Sweet Sally above, many will think that Ernie is not responsible for A, or, at least, that he is less responsible for A-ing than Bernie.^6^ In this sort of case, the victim does not act on the basis of attitudes acquired or modified via bypassing. Thus, a bypassing view does not have the resources to account for the claim that Ernie lacks responsibility.^7^

Manipulator-focused views are well-positioned to account for Ernie’s lack of (full) responsibility.^8^ Roughly, such views hold that the reason that the manipulee lacks (full) responsibility is because they were manipulated by another agent. Such views, like bypassing views, tend to have the resources to account for the claim that a subject of manipulation in a mid-life case (e.g., Sweet Sally) is not responsible for the relevant action. Unlike bypassing views, they also tend to have the resources to account for the claim an agent like Ernie is not responsible for his action. However, and also unlike bypassing views, manipulator-focused views tend to have difficulty in accounting for the claim that agents in standard natural force cases are not responsible for the relevant actions, given that such cases lack a manipulator. In fact, proponents of such views are often happy to accept this result.

Manipulator-focused views are the main topic of this paper. There are, by now, a variety of such views on offer, though some authors have only hinted at manipulator-focused views without offering developed proposals. It is worth mentioning these hints before moving on to the more robust manipulator-focused proposals on offer.

Daniel Dennett and Bruce Waller suggest that at the very least, the presence of the manipulator is affecting our intuitions (Dennett, 1984: 8; B. N. Waller, 1988: 156). William Lycan suggests that a difference between manipulated and unmanipulated agents concerns the fact that manipulated agents are puppets (1987: 117), and Jennan Ismael similarly states that the manipulated agent in such cases is “merely a puppet, an extension, or an automaton” carrying out the manipulator’s will (2016: 104). C.P. Ragland argues that intentional manipulation rules out responsibility (2011: 68), and Markus Schlosser argues that Ernie is different from Bernie insofar as Ernie’s zygote was “rigged to interact with the circumstances” so that he ends up A-ing (2015: 80). Samantha Seybold argues that in order to be responsible for some action, one must not be subject to someone who has complete control over everything one does (2022), and Xiaofei Liu argues for a manipulator-focused view more generally (2022).^9^

While all of these authors seem to adopt a manipulator-focused view, they do not provide significant detail beyond the above, apparently relying on intuitive understandings of various notions (e.g., someone’s being another’s puppet, or being under the complete control of another). Yet there are more detailed approaches. In fact, most recent new attempts to account for our intuitions about manipulated agents develop more detailed manipulator-focused views.

Our main aim in this paper is to provide a general assessment of this approach. We evaluate particular versions of such views—accounts which may be seen as extensions or developments of the ideas expressed above—in order to assess the promise that this sort of approach may have, when fully developed. We will consider an initial version developed by Gideon Yaffe (2003) in Sect. 2, variations by Robyn Repko Waller (2014) and Eric Christian Barnes (2015) in Sect. 3, a proposal by Marcela Herdova (2021) in Sect. 4, and one by Oisín Deery and Eddy Nahmias (2017) in Sect. 5. In addition to highlighting certain commonalities between this apparently disparate set of views, this paper raises several new challenges for manipulator-focused views—challenges that go beyond worries about standard natural force cases—and concludes with some general reflections on the prospects for manipulator-focused views in light of the challenges raised here.

## Yaffe

Before explaining Yaffe’s view, it is worth pointing out that, though Yaffe does briefly discuss the general debate concerning the threat that manipulated agents pose to various views of freedom and responsibility (2003: 337–340), he does not engage with the sorts of cases we gave above—*Sweet Sally*, *Natalie*, and *Ernie*—which, for now, we can call the standard cases. Yaffe seems to be more concerned with cases that are more down to earth; cases in which the manipulators are relatively normal humans—and thus lack the robust capacities of manipulators in standard cases—and in which the process of manipulation is more protracted, and not sudden. Consider, for instance, one of his main cases:

So, imagine a Kaspar Hauser-type person who is confined to a dark room from a very early age by someone determined to make him entirely docile and not prone to conduct aimed at overthrowing a particular monarch. The relevant Unlucky agent is someone who is similarly confined, and similarly conditioned, but not by a person who is aiming to condition him in this way but merely through misfortune of some sort. (2003: 344)

Given that Yaffe’s approach is mainly to explain why manipulation undermines freedom in particular cases of this sort, it is difficult to see how the view applies to the standard cases. We elaborate on this below.^10^

According to Yaffe, manipulation of the relevant sort (indoctrination) “undermines freedom by providing stricter limits on the agent’s pattern of response to reasons than are placed on the agent by neutral causal forces that cause her to respond to reasons as she does” (2003: 348). To unpack this claim, it will be helpful to consider Yaffe’s comparison of a manipulated agent (“the Manipulated”) with the Unlucky:

The crucial fact about a manipulator who aims to produce in you a certain pattern of response to reasons is that he tracks the production in you of that pattern of response. It is true of the Manipulated, and not of the Unlucky, that were the Manipulated to stray in some way or another from coming to have dispositions to recognize and respond to reasons of the sort that the manipulator wants her to have, he would take steps to see to it that she was placed back on course. The Unlucky, on the other hand, would simply stray from the course and come to have a different pattern of response to reasons from that of the Manipulated. (2003: 343-344)

On such a view, an important difference between the Manipulated and the Unlucky concerns the fact that manipulators “limit our options in ways that neutral causal forces do not” (2003: 344), and for agents subject to such manipulators, “fewer lives are available” (2003: 345). The manipulator achieves this result by tracking the production, in the manipulee, of the pattern of response to reasons.

As the case of Unlucky shows, such a view cannot account for the claim that agents like Natalie lack (full) responsibility for their actions,^11^ but it can help to account for many of the cases Yaffe considers. However, on some ways of filling out the view, it remains open to at least two concerns one might have when developing a manipulator-focused view. Consideration of these can also help to frame our discussion of more recent views.^12^

One feature of the sorts of manipulators that Yaffe discusses is that they are *online manipulators*. Manipulators that are online (a) monitor the Manipulated after the initial intervention, (b) are ready to intervene, were the agent to stray from the intended path, and (c) could (at least somewhat reliably) succeed in bringing the Manipulated back on the intended path, were they to intervene.^13^ These are three features that mark a difference between Manipulated and Unlucky. It is worth noting here that a manipulator can have these features, and thus be online, even if the victim never strays from the path, there is no need for (further) intervention, and thus the manipulator does not actually intervene.

If a manipulator’s being online is essential to the explanation of why the Manipulated lacks (full) responsibility, then the view will face issues.^14^ No such monitoring is involved in standard cases of manipulation, like that of Sweet Sally—cases in which it is just as intuitive that the victim lacks freedom as it is intuitive that the victims of online manipulators lack freedom. In fact, whether it is a mid-life case or an original design case, we can imagine that the manipulators die immediately upon implanting the new values or creating the zygote. Dead manipulators, in our estimation, tend to be offline. Yet, despite the disappearance of the manipulators from the scene, it remains intuitive that their victims lack freedom.

One might think, however, that in these standard cases the manipulators have more robust capacities. Importantly, they have more robust capacities, and they can initially—when performing the surgery, or modifying the zygote—implement a variety of plans with substantial knowledge of how they will affect future events. With these powers, one might not need to worry so much about the victim “straying from the path;” the plan would have taken into account potential causes of this and the intervention modified accordingly. And if some mishap were to occur during the surgery, or modification of the zygote, the manipulator could have fixed it. Such a manipulator could still put strict limits on the lives available to the victim without needing to be online; the relevant “tracking” occurred when planning the intervention, and even if they die right after their initial intervention, their work is done.

Perhaps, then, a manipulator’s being online is relevant to the explanation when the manipulator lacks the robust capacities of manipulators in the standard cases, yet it is not necessary with respect to the more powerful manipulators in standard cases. And, perhaps, Yaffe’s explanation could be amended to reflect this. We return to this later on (Sect. 5), where we consider what we call interventionist views, which may offer some means for making this amendment.

A second worry that manipulator-focused views need to defend against concerns a class of cases that have come to be known as “Frankfurt cases” due to Harry Frankfurt’s classic presentation of such cases in Frankfurt (1969). In a typical Frankfurt case, a neuroscientist secretly monitors the brain processes of Frank and can intervene to make Frank make a certain choice if Frank is not going to make it on his own (and the neuroscientist is a reliable predictor of such things). As it happens, Frank makes the choice the neuroscientist wants him to make without any intervention. Given the neuroscientist’s presence, it looks as though Frank cannot do otherwise than make the choice he actually makes. Yet it also looks as though Frank is responsible for making the choice. After all, Frank would have deliberated and made the choice in exactly the same way even if the neuroscientist had not been monitoring. Not everyone is convinced, of course—this is philosophy, after all—but many have taken this sort of case to show that an agent can be responsible for an action despite lacking the ability to do otherwise than that action.

The potential concern regarding Frankfurt cases arises from the fact that the would-be intervener in such a case shares various features with *actual* interveners in standard manipulation cases. The would-be intervener monitors the would-be victim, stands ready to intervene were they to stray from the path, and could reliably succeed in doing so. And because of this, Frank shares an important feature with victims in standard manipulation cases; for Frank, fewer lives are available. Consequently, we suggest, these features cannot, at least not on their own, explain why victims of manipulation lack (full) responsibility for the relevant actions. There is a relatively straightforward fix to this, which is to suggest a further relevant feature: that the intervener *actually* intervened on the agent. As we will see shortly, this is what is explicitly included in some more recent views.^15^

## Waller and Barnes

More recently, and in light of original design cases in particular, Waller (2014) and Barnes (2015) have developed manipulator-focused views that are similar to each other in important respects. Before evaluating them jointly, we introduce them individually.

### Waller

Waller’s key claim is that the effective intention of a manipulator can mark a relevant difference between manipulated agents and non-manipulated but determined agents. An intention’s being effective can be understood in multiple ways, although, as we read her, she does not commit to one understanding or another:

“Effective intention to *A*” in a deterministic universe at least entails that *A* occurs. One can also adopt a stronger reading…: if an agent *S* has an effective intention to *A* in a deterministic universe, this entails that…*S* intentionally performs that intended action *A*. (2014: 212–13)

As we interpret Waller, one difference between the strong and weak readings of “effective intention to A” is that, on the weak reading, an intention to A can be effective even if it brings about A in a causally deviant way, whereas on the strong reading, such an intention would not be effective. This difference will be relevant below. Yet on either reading, we can see how the view can mark a difference between Ernie and Bernie. Even though Bernie is identical to Ernie at every moment following the creation of his zygote, including his *A*-ing 30 years later, Bernie’s *A*-ing did not result from some other agent’s effective intention.

In light of a case like the Frankfurt case introduced above, with no intervention by the would-be manipulator (2014: 215), Waller suggests that it is not enough, to mitigate responsibility, for the intention to be effective; the manipulator needs to *actually* intervene. Fully stated, her view is:

T*: *S* is less deserving of blame or praise for *A*-ing than she would be otherwise if

1. another agent *G* effectively intends that *S A*-s,
2. *G* brings it about that *S A*-s via intervention, and
3. *S* did not intentionally bring it about that *G* intends that *S A*-s. (2014: 216)

Note that T* only claims that manipulated agents are less deserving of blame or praise, not that they lack responsibility. Still, according to T*, there is a relevant difference between Ernie and Bernie, since Diana effectively intends—via some intervention—that Ernie *A*-s (and Ernie did not bring it about that Diana intends that he *A*-s) as a result of her modifying the zygote. Further, given that the psychologists intend that Sweet Sally kill George, this view would imply that Sally is at least less responsible for the killing than a non-manipulated counterpart. However, the view cannot say the same for subjects of natural forces.

Before turning to Barnes’s view, it is worth stressing one distinct feature of Waller’s view and a potential problem it invites. Consider again the case of Carl, which involved fairly mild manipulation. On Waller’s view, Carl is less deserving of blame for deciding to eat the snack in response to the manipulator-induced urge than he is when he acts on an identical urge he acquired in the normal way (assuming he deserves any blame for either case).^16^ This point expands to ordinary interactions. If Carl’s friend suggests that he eat a snickers, given that he has recently been cranky, and Carl takes him up on that suggestion, then according to T*, Carl is less deserving of blame for eating the snickers. One might find this implausible, in which case, one may have some reason to reject the view. This may not be a strong reason, and as Waller points out (p. 215), the claim that Carl is less responsible is consistent with there being a very small difference in terms of responsibility.

However, even if Carl is *slightly* less responsible than he would have been otherwise, we suspect that a common intuition about Ernie is that he is *much* less responsible than Bernie is for the same action—perhaps even that Ernie is not responsible *at all*. Yet T* has no resources for distinguishing between Carl and Ernie; both actions meet 1–3 of T*.^17^ To make this point clearer, suppose that Ernie and Bernie perform their respective actions partly due to a suggestion from a friend (which Diana foresaw in the case of Ernie). Now Ernie and Bernie both meet 1–3 of T*, and Waller’s view does not have the resources for distinguishing between them, despite the common intuition that Ernie is much less responsible than Bernie.^18^

### Barnes

Barnes also begins with a focus on Ernie and Bernie, and, like Waller, argues that the difference will concern Diana’s intentions. Although Barnes mostly focuses on freedom rather than responsibility, he is thinking about both, and his key claim is that, while freedom (and responsibility) requires creativity (understood in a certain way), manipulation mitigates creativity.^19^ Barnes proposes that “the existence of nearby possible worlds in which agents perform creative acts…is a necessary condition for an agent’s being fully free” (2015: 567).^20^

What does it mean to perform a creative act? Barnes begins with the notion of an idea’s being creative: “I propose to count as a creative idea an idea that an individual has not acquired because his community communicated the idea to him—such an idea [that has not been acquired because it was communicated by one’s community] would count as instantiating ‘communal creativity’” (2015: 568). From there, Barnes builds up to the notion of acting creatively:

I require that for an agent to perform action A (which may be a thought) freely that there be at least one nearby possible world (which may be the actual world) in which he exhibits communal creativity by acting in a way which his community has not communicated to him. I will deem this the Potential Creativity Condition (PCC) for freedom.^21^

I offer the following clarification of what it means for a community to communicate an action X to B: (1) A (a member(s) of B’s community) grasps the idea of X and (2) intends for B to grasp the idea of X and (3) performs actions that serve to transmit information constituting the idea of X from A to B. (2015: 568)

Barnes (560–1) then considers a variant of the Ernie case (though he intends for his view to apply to the original version as well (560–1)) and says that Diana counts as part of Ernie’s community because “she is a person who is engaged in the direct shaping of [his] cognitive and behavioral development” (2015: 572). Further, she grasps the idea of his A-ing (1), and intends for Ernie to grasp that idea (2). It is not clear to us how it is that Diana transmits the information to Ernie (3) in the original case—that is, it is not clear to us how she communicates the idea (or anything) to Ernie by modifying his zygote—but for our purposes, we grant that she does.^22^ If this is right, then although Bernie meets PCC, Ernie does not; while Bernie freely *A*-s, Ernie is not (fully) free with respect to his *A*-ing. If we grant this, then we could offer a similar explanation for Sweet Sally’s lack of (full) responsibility as well.

Further, Barnes’s view can avoid getting the wrong verdict with respect to Frankfurt agents. Since would-be interveners in Frankfurt cases don’t actually intervene, they don’t “perform actions that serve to transmit information constituting the idea of X from A to B” (3 above).

### Evaluating Waller’s and Barnes’s views

Both Waller’s and Barnes’s views avoid issues with Frankfurt cases, insofar as their conditions involve the claims that the manipulator either intervenes on, or communicates the action to, the manipulee and neither happens in a Frankfurt case. And since neither requires the manipulator to be online, they can accommodate dead manipulator variations as well. Further, Waller’s and Barnes’s views are similar in that they apply to the manipulee’s action that was intended by the manipulator, where the intention was effective via intervention, or was communicated.

Because of this reliance on intention, they cannot account for the claim that agents in standard natural force cases—like Natalie—are not (fully) responsible. But further, it opens up a different challenge. One way to make this vivid is by considering variations of a manipulation case which we might call *accidental results cases*. Consider three variations of the case of Sweet Sally:

*Extra Killing* Sally notices that her other neighbor, Henry, saw her dispose of George’s body. Since post-manipulation Sally is the sort of person that leaves no witness, she also kills Henry. As in the killing of George, this killing would have been unthinkable to Sally before her manipulation. Yet, in this case, the team of psychologists only developed their plan up to the point of Sally’s killing George and could not have foreseen, and did not intend, the additional killing.

*Wrong Killing* Sally’s manipulators are not very good at what they do, and while they come very close to succeeding at getting Sally to murder George on the basis of her new psychological profile, she kills her other neighbor Henry instead.

*Wrong Manipulee* Here the manipulators *are* good at what they do, and have elaborate plans for Hallie, beyond her murdering her neighbor, Steve. But they were given the wrong address. Intending to manipulate Hallie, they accidentally manipulate Sally instead, and Sally murders George.

We suspect that if one thought Sally was not responsible for killing George in the original *Sweet Sally* case, one will not think she is responsible for the murder(s) in each of these cases. Yet, the manipulators did not foresee, much less intend, that Sally perform these murders. None of this behavior lacks creativity, in Barnes’s sense, since the manipulators did not communicate these actions to her (in virtue of failing to meet (2) above). Nor is Sally’s behavior in these cases effectively intended by the manipulator (or any agent other than Sally). Thus, neither Waller’s nor Barnes’s view can accommodate the claim that these Sallys lack (full) responsibility.

One initially tempting remedy may be to somewhat loosen the relationship between what the manipulator intends, and what the manipulee lacks (full) responsibility for. For instance, keeping the other components of the views fixed, one might suggest something along the lines of: “the better that the action fits the content of the manipulator’s intention, the less responsible that the agent is for the action.”^23^ In *Extra Killing*, for example, the manipulator does not intend for Sally to kill Henry, yet the killing does, in some sense, fit the content of their intention; it is an instance of Sally killing. On this amended view, Sally may have mitigated responsibility for killing Henry; though perhaps more than she did for killing George, given that the latter action fits the content even better. Similar points apply to Sally’s murder in *Wrong Killing*.

Yet we think that, even if such an amendment could be implemented to both of these views, a solution along these lines still faces further issues. For instance, in *Wrong Manipulee*, it is both the case that Sally is the wrong manipulee and that she kills the wrong person. But we think she lacks (full) responsibility for the killing, and as much as (the original) Sweet Sally. This view would suggest otherwise. Further, consider variations in which agents perform a different sort of action than what was intended; e.g., Sally’s burying George’s and Henry’s bodies in *Extra Killing*. Or, for instance, suppose the manipulators had intended for their target to bake a nice pie for their neighbor, yet due to a mistake, they end up with the same results as above: Sally’s committing murder.

## Herdova

Herdova (2021) argues that, in virtue of the fact that Ernie is Diana’s tool, he is neither free nor responsible for A-ing. More specifically, she argues that “being a tool may either take one’s freedom or responsibility altogether or at least significantly diminish it, if enough of the toolhood contributing elements are present” (2021: 261). Herdova’s approach to an account of toolhood is not in a traditional form; i.e., she does not present explicit necessary and/or sufficient conditions for A’s being a tool of B. Rather, she offers a non-exhaustive list of some typical elements of A’s being a tool of B:

- B intentionally, covertly and non-consensually ensures that A acts in certain ways, and does all this not merely by omission.
- B has a vested interest in A acting in these ways.
- A would not have acted in those ways, nor even had the psychological profile he has (and, in some cases, would not even have existed), had B not intentionally brought it about that he does.
- Had B wanted A to do or think something else, A would have done so.
- In ensuring that A acts in certain ways, B intentionally bypasses A’s rational faculties, or B sees to it that A is in a situation whereby his rational faculties recommend only one possible course of action, or B’s actions give rise to exactly the right rational faculties in A to suit B’s own ends (or all or some of the above).
- The relationship is not reciprocal: A does not make B behave the way he does or have the psychological profile he does, or even allow B to do what he does. (2021: 258)

On this account, “when enough of these conditions are present, they can collectively contribute to the extent to which someone is the tool of another” (2021: 259).^24^ In the case of Ernie, all of (a–f) are met, and thus, Ernie is Diana’s tool. Similar things apply to the case of Sweet Sally.

Although this view is somewhat different from those previously discussed, both in content and in form, it faces some of the same issues. First, notice that none of (a–f) apply in a standard natural force case, since there is no manipulator (and thus no B) in such cases.

Second, consider *Wrong Killing*. In this case, the manipulator has no vested interest in Sally’s killing Henry (b). Further, it is not clear that the manipulation constitutes an intentional ensuring that Sally kills Henry (a); and for similar reasons, the manipulator does not intentionally bring it about that Sally kills Henry (c). Nor does it seem to be true that, had the manipulator wanted Sally to do something other than kill Henry, she would have done so (d), since the manipulator *did* want Sally to do something else. Similar points can be made about *Wrong Manipulee*: (a), (b), and (e) do not apply, and arguably (d) does not either. Thus, it is not clear whether this view would imply that these versions of Sally lack (full) responsibility for the relevant actions.

Third, recall again the case of mild manipulation. All of these elements, except for (c), apply with respect to Carl’s eating snacks. On this view, a subject of mild manipulation might be a better candidate for reduced, or eliminated, responsibility than Sally in *Wrong Killing* and *Wrong Manipulee*.

Finally, consider *Extra Killing*. Sally is intuitively the manipulator’s tool when she kills George—the intended target—and Herdova’s account gets the right result, insofar as, with respect to this action, all of (a–f) apply. However, the manipulator does not intentionally ensure that Sally kills her witnessing neighbor, *Henry*, insofar as this was not part of the manipulator’s plan, and we can stipulate that the manipulator would not have modified her intervention, were she to realize that it would result in Sally’s not killing Henry. Thus, (a), and for the same reason (e), do not apply to this action. Nor is it the case that the manipulator has a vested interest in Sally’s killing Henry (b). Since the manipulator does not intentionally bring it about that Sally kills Henry, (c) would not apply either. By our estimation, at most only (d) and (f) apply to this action; perhaps, had the manipulator wanted Sally to do something else, she would have done so, and the relationship is not reciprocal. Notice, however, that (d) and (f) are also true of agents in Frankfurt cases. Thus, either these two are enough to at least mitigate Sally’s responsibility for killing Henry, in which case Frankfurt agents *also* have mitigated responsibility. Or, (d) and (f) are not enough to mitigate responsibility, in which case the view cannot accommodate the claim that this version of Sally is not (fully) responsible for killing Henry.

## Interventionist views

We turn now to a family of views which rely on a particular account of causation (Deery & Nahmias, 2017; Murray & Lombrozo, 2017; Usher, 2020). In particular, they implement resources from *interventionist theories of causation* (Hitchcock, 2001; Woodward, 2003, 2006) in order to assess manipulation cases. Roughly put, such views evaluate causal claims relating three variables—the outcome to be assessed, the potential cause, and the background conditions—by seeing what would happen to the outcome variable when changes are made to the other variables. For reasons of space, we focus on the view developed by Deery and Nahmias (DN), though much of what we say applies to these other views as well.^25^ And since these views of causation are technical and complicated, we opt for a rough sketch of such views, while brushing over much of the detail.^26^

### Deery and Nahmias

DN intend to offer an account of causal sourcehood which can be used to solve a variety of issues related to free will and moral responsibility. Thus, though they mainly show how their account would apply to the case of Ernie—a case of original design—they do not suggest that this view is justified solely by its ability to account for the claim that some manipulated agents lack (full) responsibility.^27^ Accordingly, though our evaluation will pose problems for this account’s ability to respond to cases of manipulated agents, we do not think they clearly pose a problem for this as an account of causal sourcehood.

Interventionism makes use of causal models that can help to illuminate the relationships between various causal variables and their effect(s). To build a model for a case like that of Ernie, we set a variable for the output of Ernie’s Compatibilist-friendly Agential Structure (CAS)—i.e., his decision to A—which we can call *X*, and a variable for the subsequent action—Ernie’s A-ing—which we can call *Y*.^28^ To test whether Ernie’s decision is a cause of the subsequent action, we begin by making an intervention on *X*; that is, we set a value for *X*, without making a change to any of the usual causes of *X* nor to the background conditions. We then see if the value of *Y* changes.^29^ On this view, “*X* causes *Y* just in case, for at least some state of the model, there is an intervention on *X* that would reliably change the value of *Y*” (2017: 1261).

This feature can come in degrees, depending on how well changes in values of *X* predict changes in values of *Y*. Tierney and Glick helpfully label this feature of causal relations *reliability* (2020: 959). Suppose a world-class dart thrower decides that she will shoot for the triple-twenty, shoots, and hits the target. Given that she is very good at this, a different decision, say, to shoot for the bullseye—a different value of a correlate of *X*—would be a good predictor of the outcome of the throw—a different value of a correlate of *Y*. This is very different from the relationship between, say, one of our decisions to aim for a particular spot on the board and the outcomes of our throw.^30^

A second feature of causal relations is what Tierney and Glick refer to as *stability* (2020: 959). To borrow a case from Marius Usher, compare shooting a person in the leg to shooting a person in the heart, when there is no medical help readily available in either case (2020: 308–9). Both shootings, we can suppose, cause the victims’ deaths. Yet shooting a person in the leg is more sensitive to changes in background conditions than shooting them in the heart, and this is because there are many more ways of changing the background conditions—e.g., the availability of medical help—which can change whether the victim dies. The causal relationship between shooting the victim in the heart and the victim’s death is more stable than that between shooting another victim in the leg and their death.

According to DN, in order to identify the causal source of an event, “we need to identify the causal variable that has the most stable causal-explanatory relation with the effect variable” (2017: 1261); where by “stable,” DN seem to have *both* reliability and stability, as described above, in mind. Thus, they develop an account of (relative) strength of invariance:

A causal invariance relation, *R*₁, that obtains between two causal variables, *X* and *Y*, is stronger than another such relation, *R*₂, obtaining between *Y* and another of its prior causal variables—for instance, *W*—iff:

1. Holding fixed the relevant background conditions, *C*, *R*₁ predicts the value of *Y* under a wider range of interventions on *X* than *R*₂ does under interventions on *W*; and

2. *R*₁ predicts the value of *Y* across a wider range of relevant changes to the values of *C* than *R*₂ does. (2017:1262–3)

The first condition states that *R*₁ is a more reliable causal relation than *R*₂, and the second states that *R*₁ is a more stable causal relation than *R*₂. This notion of (relative) strength of invariance is the foundation of their account of causal sourcehood:

*X* = *x* is the causal source of *Y* = *y* iff *X* bears the strongest causal invariance relation to *Y* among all the prior causal variables (including *X*) that bear such relationships to *Y*. (2017: 1263)

This relates to manipulated agents insofar as, on DN’s view, whether an action has its causal source in the agent or not can affect their free will and moral responsibility. DN suggest that, when it comes to freedom and responsibility with respect to some action, their view “adds the requirement that the agent’s action have its causal source in the agent’s CAS” (2017: 1267). As we read this, and other claims in the same context, it is a necessary condition on an agent’s *being* responsible for some action. However, they sometimes speak of responsibility being *mitigated,* and elsewhere say that “[w]e do not take a stand on whether [Ernie] lacks free will entirely or whether he has less or a lesser sort than [Bernie]” (2017: 1260, n. 6).^31^ Thus, an alternative interpretation would suggest that this is a requirement on an agent’s being *fully* free or responsible for some action, where “fully” is understood as something like “as free or responsible as a standard agent.”^32^

With this view in hand, they go on to show why Ernie lacks (full) responsibility for A-ing. This is because the causal source of Ernie’s A-ing lies in Diana, not in Ernie; Diana’s decision that Ernie A bears a stronger causal invariance relation to Ernie’s A-ing than Ernie’s decision. Consider the fact that, “[h]ad Diana decided that she wanted [Ernie] to [do some B other than A], then she would have created his zygote in a different way so that he would decide to [B]” (2017: 1265). Further, since Diana can ensure that Ernie will A, “there are no relevant changes to conditions *C* that could possibly interfere with [Ernie’s A-ing], since Diana…has foreseen all such possibilities” (2017: 1265). In this respect, and with respect to condition 2 above, the relation between Diana’s decision and Ernie’s A-ing wins out over the relation between Ernie’s decision and Ernie’s A-ing; it is a more stable relation.^33^

To illustrate, DN offer a case. Suppose that we make a slight modification to the background conditions, and after Ernie decides to A, and just before he begins to A, he receives a text from his sister telling him that she has landed on Mars. Filled with pride in her achievement, he forgets about A-ing and walks away. At the time that he sees the text, “it is nomologically impossible for activity in [his] CAS to produce an output that controls for the influence that [the] message has on whether he steals (he cannot know about the message before it arrives)” (2017: 1266). Yet, since Diana is able to foresee everything that might affect Ernie’s action, she would have foreseen this, and changed her modification of the zygote accordingly. That is, in this change of background conditions, the relation between the output of Ernie’s CAS and his action is not as predictive as the relation between Diana’s decision and Ernie’s action. And presumably, there are many such cases.

Thus, Diana’s decision is the causal source of Ernie’s action, as opposed to his own decision. In the case of a standard agent like Bernie, however, we can suppose there is no such preceding cause, and his decision is the causal source of his action. We suspect that we can tell a similar story with respect to the difference between Chuck and the original case of Sweet Sally.

### Evaluating interventionist views

Interventionist views of causation, and the corresponding manipulator-focused views, are highly intricate. Assessing causal claims in general, and causal sourcehood in particular, involves the evaluation of a large variety of factors, and it will often be difficult to know how such views apply to particular cases.

Yet this is not impossible, since we can point to some features of causal relations, events, or processes that may be, at least, indicators of stability and reliability. For example, although both Diana’s and Ernie’s decisions are relatively stably and reliably related to Ernie’s action, it is also the case that Diana (a) knows how different interventions will play out, given the laws and a specified set of states of the universe, (b) has the ability to implement these various interventions, and (c) intends for her intervention to result in Ernie’s A-ing 30 years in the future. Thus, it seems that the causal source of Ernie’s action lies outside of Ernie. Similarly, these features seem sufficient to allow Diana to “track” Ernie’s pattern of response to reasons, in the sense Yaffe seems to have in mind. Given these similarities, we will occasionally bring up Yaffe’s view when relevant.

An initial problem for interventionist views is that not all of these features are present in our accidental results cases.^34^ They all lack (c), for example. In *Wrong Killing*, the manipulator does not intend for Sally to kill Henry; in *Wrong Manipulee*, the intention is to manipulate a different person, not for Sally to kill George; and, in *Extra Killing*, the manipulator does not intend for Sally to *also* kill Henry. Further, they do not even have the knowledge (a) that their interventions will have such results. In fact, in *Wrong Manipulee* and *Wrong Killing*, had they known what their interventions would actually result in, they would have acted otherwise. And if the manipulator lacks the relevant intention, as well as the knowledge of the results of their interventions, it is not clear why the ability (b), if they have this, matters with respect to stability and reliability. Because of these differences between our cases and the paradigmatic cases of Ernie and Sweet Sally, interventionist views may not be able to account for the claim that these versions of Sally are not (fully) responsible.^35^

We suspect that we can motivate the problematic nature of these cases by adding substantial luck somewhere or other. For instance, perhaps most slight changes to background conditions would make it so that either Henry, in *Extra Killing*, does not pass by Sally’s window at just the right time, or Sally does not notice Henry passing by the window. In such cases, Sally does not kill him. But even supposing interventionist views can handle the accidental results cases discussed above, we now turn to some cases where it is clearer how to apply these views and, we argue, interventionist views get the wrong results.^36^

With respect to Yaffe’s view, such cases also become somewhat complicated. We find it plausible that the manipulators are not tracking Sally’s pattern of response to reasons in *Wrong Manipulee*. Yet, given that they intended to manipulate Sally in *Wrong Killing* and *Extra Killing*, and given that the *sorts* of reasons relevant to these actions, though perhaps not the *particular* ones, are of the kind they intended to make Sally responsive to, a view like Yaffe’s may be able to account for their lack of (full) responsibility. That is, it is plausible that, despite the fact that the manipulators did not intend for their respective Sallys to perform the particular relevant action, they may well have tracked the pattern of response to reasons relevant to her doing so.^37^

We can now consider a new set of cases.

#### Lucky manipulators

First, we offer a further variation of Sally. In order to become the expert manipulators that we find in standard cases, our manipulators went through a process of acquiring the relevant skills. So, consider:

*Fledgling Neurosurgeon* 20 years before he manipulated Sally, our Fledgling Neurosurgeon decided to begin to learn how to perform this sort of surgery. He begins by trying to turn an equally sweet person, Nelida, into someone as evil as post-manipulation Sally. It works: Nelida wakes up, reflects on her new values, and decides to kill Juan, her neighbor. When he tries to do this again on other subjects, he fails miserably. Most of them die on the operating table, and the rest end up with strange results: an obsession with petting penguins, feeling extreme pangs of pain every time she sees corduroy, or thinking of oneself as a kite floating over the Sahara. His success with Nelida was not a pure stroke of genius, but rather a pure stroke of luck.^38^

We suspect that, if one thought that Sally was not (fully) responsible for killing George, one will similarly judge that Nelida is not (fully) responsible for killing Juan. Can interventionist views accommodate this? We do not see how. Notice that, due to Fledgling Neurosurgeon’s lack of skill, it is likely that had his intention been slightly different—that Nelida kill someone else, say—or the background conditions been slightly different, then he would not have succeeded. However, due to the results of the surgery, Nelida, like Sally, retains the relevant agential qualities; her decision to kill Juan is the result of adequate deliberation, and her killing of Juan is intentional and skilled (suppose she was an excellent shooter). Nelida’s decision bears a stronger causal invariance relation (in DN’s sense) to Nelida’s action than does Fledgling Neurosurgeon’s decision to manipulate Nelida. Similarly, given Fledgling Neurosurgeon's  lack of skill, he seems to lack control over Nelida’s pattern of response to reasons, and so does not “track” it in the relevant sense.^39^ We pause briefly to point out that this case is not a problem for all manipulator-focused views. Insofar as Waller requires an effective intention, yet stops short of requiring that the intention be effective via *intentional* action, her view would suggest that Nelida’s responsibility is, at least, mitigated.^40^

Now consider another pair of cases involving luck that present a different sort of problem. Here is the first:

*Random Machine Induction* Mac has a machine that can, by sending certain powerful and terribly precise radio-signals, make similar modifications to a victim’s value schemes as do the psychologists in the Sweet Sally case. Mac is transporting this machine on his way to his next target, and he has already pre-programmed the signal it will send. Unbeknownst to him, the machine is on and ready to send its signals; all that is needed is for it to be aimed and the button to be pressed. While he is checking his GPS, he distractedly drives onto a rumble strip on the side of the road, which causes the coffee cup he had left on top of the machine to fall and hit the button. Unluckily for Sweet Claudia, the machine was, at that moment, perfectly aimed to modify her values as she slept on the bus next to Mac’s truck. When she arrives home, she wakes up, reflects on her new values for a bit, and kills Pablo, her neighbor.

DN’s view seems best suited to get the right result in a case like this, where the manipulator lacks an intention that the manipulee act in a certain way, but even their view won’t work here. Because the machine’s signals are very precise, and the chain of events leading to the signal being fired are quite (un)lucky, relatively small changes in these events—the cup falling a split-second later, or a centimeter to the left, etc.—will not result in this radical change in Claudia. And because the machine needs to be aimed very precisely, relatively small changes to the background conditions—e.g., the relative speed of the truck and Claudia’s bus, the relative locations of these vehicles, etc.—will also avoid this result. Thus, given that Claudia’s decision and action issue from her CAS, we suspect that the causal source of the action is her decision.

This case also lays the foundation for a further issue. Consider:

*Lucky Machine Induction* Everything is the same as in Random Machine Induction except that, in this case, Mac’s target was Claudia, whom he intended to manipulate in the same way, once he got to her house. Once the machine sends its signal, Mac receives a notification on his phone that it has done so. He then finds out that the signal was directed at Claudia, and gathers that she will end up killing Pablo. Since he does not need to do anything further, he sits back and waits. Claudia goes on to kill Pablo.

We suspect that, if one thinks that Claudia is not responsible in *Random Machine Induction*, one will judge that she is not responsible in *Lucky Machine Induction* either. However, the interventionist accounts may treat these cases differently. Assuming that we include details about how the machine is activated, and the time at which it is, then the actual process by which Claudia is manipulated in this case is, like in *Random Machine Induction*, not of the sort that such accounts find problematic; it is not more of a source than Claudia’s mental states. But Mac’s *decision* to manipulate Claudia, or his *intention* to do so, do seem to change things. Of course, Mac’s intention, at best, deviantly causes the manipulation, insofar as he was implementing part of his plan to do so when checking the directions to Claudia’s house. But this does not seem to change the fact that Mac’s decision, or intention, is more of a source of Claudia’s actions than her mental states. Had anything been slightly different on the road, Claudia would not have undergone this change, while on the road. Yet Mac would have continued with this plan and used the machine on Claudia to the same effect later on.

Thus, although the particular event of sending the signal is not related to Claudia’s action in a relatively stable way, Mac’s decision, or intention, to manipulate Claudia into killing Pablo *is*, since changes to the background conditions would have, at best, resulted in his sending the signal later on. Further, had he planned for Claudia to do something else, he would have pre-programmed the machine to send a different signal. Keeping the background conditions fixed, this different signal would have been sent to Claudia on the road. Consequently, the causal relation between Mac’s decisions or intention and Claudia’s action is relatively reliable as well. We suspect many readers will agree with us in thinking that it is a strange implication of a view that it gets these different results between *Random Machine Induction* and *Lucky Machine Induction*.^41^

Yaffe’s view, we suspect, will get a similarly strange result. Given that Claudia is not Mac’s target in *Random Machine Induction*, it is doubtful that he is tracking her pattern of response to reasons in the relevant sense. Thus, his view cannot account for the claim that she lacks (full) responsibility. In *Lucky Machine Induction*, however, she is his target, and he stands ready to intervene and ensure that she exhibits a particular pattern of response to reasons. When introducing Frankfurt cases, we mentioned that there may be a relatively straightforward fix to Yaffe’s view that helps to avoid getting it wrong in the case of Frank. Namely, we suggested that Yaffe could offer a further component in the explanation requiring that the manipulator have *actually* intervened in the first place. Adding this component, however, would seem to get us the result that Mac undermines Claudia’s responsibility in the relevant way.^42^

#### Parallel cases

Finally, consider what are sometimes called *parallel cases*—cases where manipulated agents meet conditions on free action posited by at least some incompatibilist views.^43^ Consider, for instance, a variation of the original case of Sally but in which agents’ decisions are sometimes indeterministically caused.

*Parallel Sally—George.* Everything is as in the original case of Sally. However, in this case, the manipulators can only instill Chuck’s bad values, hoping that Sally will end up killing George, but they cannot get over this indeterministic hump. Just before she decides to kill George, it is open to Sally—it is consistent with the past and the laws of nature—to decide to kill his son instead. She decides to kill George and does so.

Had the manipulators not instilled Chuck’s values, neither option would have occurred to Sally. We find it plausible that Sally is not (fully) responsible for killing George. However, it is not clear how interventionist views can account for this claim, given that the indeterminacy would seem to prevent the manipulators from being more of a causal source than Sally. The output of Sally’s CAS in this case—her decision to kill George—would seem to be causally related to her killing of George in a more stable and reliable way.

We pause again briefly to note that, as with *Fledgling Neurosurgeon*, this case is also not clearly a problem for some other manipulator-focused views. Sally’s killing of George is still the result of the manipulators’ effective intention, and, according to Waller’s view, she lacks (full) responsibility.

We do think, however, that a slight variation of this case poses a problem for most other views as well. Consider, then:

*Parallel Sally—Son.* Everything is as in *Parallel Sally*, yet Sally instead decides to kill George’s son, and does so.

We suspect that, if one thought that Sally in *Parallel Sally—George* is not (fully) responsible for killing George, then one will think the same of Sally’s killing his son in this case. The difference, however, is that the manipulators do not intend for Sally to kill George’s son; the manipulators fail in their plan. In this respect, this case is like *Wrong Killing*, yet which includes indeterminacy at, or just before, the time of decision.

This variation would seem to pose problems for the interventionist accounts for the same reasons that *Parallel Sally—George* did. But given that it is also an accidental result case along the lines of *Wrong Killing*, it poses problems for views that had trouble dealing with the latter—i.e., Waller’s, Barnes’s, and possibly Herdova’s views.^44^ Yaffe’s view, on the other hand, may be able to account for both Parallel Sally’s lack of (full) responsibility, and for similar reasons as with the cases of *Extra Killing* and *Wrong Killing*. In these cases, the manipulators actually intervene and successfully induce the pattern of response to reasons that they would seem to be tracking.

## Conclusion

We have brought together a set of apparently disparate views—manipulator-focused views—and highlighted certain commonalities between them. Along the way, we have raised a set of new challenges for views of this type that go beyond the initial worry about their capability to deal with standard natural force cases. We have seen that, while some views avoid some problems that plague other views of this type, each view comes with costs.

Here we conclude with some general reflections on the discussion thus far. We wish to make clear, however, that we do not mean to suggest that the manipulator-focused approach to manipulation cases is doomed to fail. As far as we can tell, any approach to cases of manipulated agents is going to involve accepting some counterintuitive result. Yet if, when deciding which view is best, one desideratum is that they tend to avoid counterintuitive results, then our discussion can help to evaluate the extent to which views meet this desideratum.

### Problems identified for manipulator-focused views

#### Online manipulators

One feature that a manipulator-focused view might have—Usher’s does, Yaffe’s might—is that the explanation of why a manipulated agent is not (fully) responsible for the relevant action involves appealing to the fact that the manipulator is online. As we saw, there is a recipe for making any case a problem for such views: have the manipulator die right after their intervention, but before the victim’s action. As far as we can tell, requiring that the manipulator be online in order for the manipulee to lack (full) responsibility for the relevant action does not help account for any case that other views cannot account for. Thus, we think that a manipulator-focused view should not rely solely on the fact that a manipulator is online in order to explain why the manipulee is not (fully) responsible for the relevant action.

#### Manipulators’ intentions and what the manipulee is not (fully) responsible for

One feature that these views differ on concerns the relationship between the content of the manipulator’s intention, and what, in particular, the manipulee lacks (full) responsibility for. Some views posit a relatively tight connection between these two. On Waller’s view, the action that the manipulee lacks (full) responsibility for is the one that the manipulator effectively intended. On Barnes’s, it is the action that the manipulator “communicates” to the manipulee. On Herdova’s view, the connection may not be so tight, insofar as she appeals to the fact that the manipulator intends and ensures that the manipulee acts in “certain ways.” Depending on how specific we are to understand “certain ways,” there may be multiple different actions that the manipulee performs—actions which are somewhat different from what the manipulator intended—which still involve the agent acting in the relevant ways, and thus result in mitigated responsibility. As we saw, a tight connection gives rise to counterexamples of the accidental results variety—including *Parallel Sally—Son—*since these results were not intended (or communicated) by the manipulator. And even loosening the connection somewhat, for instance, in terms of how well the relevant action fits with the content of the manipulator’s intention, still faces issues (sec. 3.3).

Yaffe suggests that the relevant tracking can occur without the presence of an intention.^45^ And an interventionist view like DN’s does not explicitly appeal to the content of the manipulator’s intention. Thus it is less clear whether these sorts of cases pose problems for interventionist views. Yet we gave reasons for thinking that the fact that the relevant actions by the victims are not intended by the manipulators may result in less stable and/or reliable causal relations between the manipulators’ intentions and the manipulees’ actions. This relation may be even weaker with respect to *Wrong Killing* and *Wrong Manipulee*, in which either the murder victim, or the manipulee (or both) is *contrary* to what the manipulators intended. And, as we suggested, introducing substantial luck to these accidental results cases makes it less likely that the manipulators’ decisions, intentions, or interventions are the causal sources of the relevant actions.

#### Intentional behavior and skills

Another relevant feature concerns not the content of the manipulator’s intention, but whether the manipulator *intentionally* achieves the result, and relatedly, whether it is relevant that they have robust abilities and/or skills to implement their intention. For brevity, we simply focus on skill. Some of the features of tool-hood on Herdova’s view suggest a level of skill, given the manipulator’s *intentionally* intervening, and the focus on *ensuring* that the agent act in certain ways. And though DN do not explicitly appeal to the manipulators’ intentionally achieving their intended result, nor to their being skilled in doing so, we expect these to be importantly related to the features of causal relations that they do take to be important (and similarly for Yaffe’s tracking).^46^

Including a criterion concerning the manipulator’s skill, or their intentionally achieving their result, comes with some downsides, but also some benefits. A focus on skilled behavior gives rise to luck-based counterexamples, exemplified in our case of *Fledgling Neurosurgeon*. Though the manipulator succeeds in doing what he was trying to do, he is not skilled at this sort of procedure. Because of this, views like Herdova’s, DN’s, and Yaffe’s face difficulties with it. A view like Waller’s, on the other hand, which neither requires intentionally nor skillfully achieving the result, can avoid some of these counterexamples.

Yet, we also saw how a view like Waller’s faced an issue with mundane interactions; a friend’s suggesting some action may mitigate responsibility for that action. Further, Waller’s view did not have resources for distinguishing such a case from a case of robust manipulation, like the cases of Sally and Ernie. Given their emphasis on skill, and robust abilities to implement their intentions, a view like Yaffe’s or DN’s can plausibly distinguish between cases involving a mere suggestion from a friend—since the friend lacks these robust abilities—and a case like that of Sally and Ernie.^47^

#### Interventions, causes, and Frankfurt cases

Another concern for manipulator-focused views is that they need to be able to avoid claiming that the agent in a Frankfurt case is not (fully) responsible for choosing as he does. As we saw, the would-be intervener and Frank share features with standard manipulators and manipulees, respectively, that are taken to be relevant by a view like Yaffe’s. One might avoid this by further requiring that the manipulator actually intervenes, and Waller’s and Barnes’s views avoid it precisely because they make this stipulation. With respect to Herdova’s view, the issue was that, of the elements of toolhood identified by Herdova, the only ones that might apply to Sally’s murder of her witnessing neighbor Henry in *Extra Killing* also apply to Frank. Namely, had the (would-be) manipulator wanted the (would-be) victim to do something else, the victim would have (d), and the relationship is not reciprocal (f).^48^

#### Eliminating vs. merely mitigating responsibility

Finally, another feature to consider is whether, on a particular view, the manipulee’s responsibility for the relevant action is eliminated or merely mitigated. Both Barnes and DN sometimes say that the manipulee’s responsibility is mitigated, and sometimes that they lack responsibility. Yet Waller focuses on mere mitigation.

If one thinks that subjects in our cases are *not* responsible for the relevant actions—as is claimed when they are typically introduced—then views which focus merely on *mitigated* responsibility will only take us so far. But even if one thinks that these agents are merely *less* responsible—or, at least, one is open to this possibility—some issues remain. This was clearest with Waller’s view, which does not have the resources for distinguishing between the, perhaps barely, mitigated responsibility of a subject of mild manipulation like Carl, and the, perhaps very substantially, mitigated responsibility for an agent like Ernie or Sally.^49^

### A return to the overall dialectic

As we mentioned early on, an alternative approach to manipulation cases explains the effect on the manipulated agent’s responsibility by partly appealing to the fact that the manipulated agent’s capacities for control over their mental life were bypassed. Put very roughly, on such a view, an agent is not responsible for an action if it issues from attitudes that were changed via bypassing, and the bypassing resulted in a significant change. It looks like bypassing views cannot account for the claim that Ernie lacks (full) responsibility, but they can get the right result in standard natural force cases (the inverse of the results of manipulator-focused views). We think that a bypassing view can account for the claim that manipulated agents in the other cases discussed above lack responsibility, though they face further issues elsewhere.^50^

Since bypassing views do not require any manipulator whatsoever, they don’t face challenges from cases in which the manipulator is offline, in which the manipulator gets lucky, in which the manipulator’s intentions are not effective, or in which the manipulee’s responsibility is affected for an action not included in the content of the manipulator’s intention. And whether the manipulee is the wrong one, whether they kill the wrong person, or whether they end up killing an extra person, do not affect whether the manipulee kills on the basis of attitudes changed via bypassing that resulted in a significant change. Consequently, such views don’t face issues with the cases we introduced. Bypassing views can, for similar reasons, account for the intuition that Sally lacks responsibility in both versions of *Parallel Sally*. Additionally, since a Frankfurt intervener does nothing to alter the attitudes from which Frank acts (much less alter them via bypassing), bypassing views are not threatened by such cases.

All of these views—both bypassing and manipulator-focused views—are presented as, at least partly, justified by their ability to accommodate our intuitions about particular cases of manipulation. Although some of the original proponents of bypassing views have continued to refine their views over the years (Fischer, 2012, Chapter 11; Haji & Cuypers, 2008; McKenna, 2016; Mele, 2019), it seems to us that most new attempts to accommodate our intuitions about manipulated agents’ lack of (full) responsibility are manipulator-focused views. We suspect that this has something to do with the fact that bypassing views cannot account for agents’ lack of (full) responsibility in original design cases, like the case of Ernie. However, even if we set aside standard natural force cases, there are a variety of other cases that speak in favor of a bypassing view over a manipulator-focused view. This is not to say that manipulator-focused views face more problems than bypassing views, only that there are other cases that pose problems for manipulator-focused views but not for bypassing views. Thus, bypassing accounts remain a live option, and the cases that favor such views over manipulator-focused views are much more varied than standard natural force cases.^51^

As things stand, every existing view needs to accept some counterintuitive conclusion. This is, of course, not to say that these are the only deciding factors, but it is all that we have the space to consider here.

## Footnotes

1 Cases like these are often set in deterministic universes and employed against the view that moral responsibility is compatible with determinism. We omit discussion of this part of the debate. At least for cases like that of Sally, there are similarly problematic variations in indeterministic settings (Clarke 2012; Cyr 2016, 2020a; Haji and Cuypers 2001; King 2013; McKenna 2016; Mele 2019: 124–6).

2 Hereafter we shorten “morally responsible” to simply “responsible.”.

3 For some discussion, see (Fischer 2012: 196–200; McKenna 2017: 579–80; Mele 2019: 35–8, 54–5, 130–3).

4 See, for instance, (Arpaly 2006; De Marco 2023b; Fischer 2012; McKenna 2017; Mele 1995).

5 Such cases are also discussed in (Barnes 2015: 564; De Marco 2023b; Deery and Nahmias 2017: 1272; Fischer 2011: 270, 2016: 50; Liu 2022; Mele 1995: 168, 2006: 141, 2019: 27, 31, 58, 94, 155 n. 10; Mickelson 2019: 171; Pereboom 1995: 25, 2001: 115, 2014: 82; Sartorio 2016: 160–1).

6 Some authors we discuss focus on freedom instead of responsibility. Given that they tend to be thinking of the sort of freedom required for responsibility, we simply speak in terms of the latter.

7 Some have argued that there is an important difference between mid-life manipulation and original design, such that it may be (Mele 2019: Chapter 4) or actually is (Fischer 2016) the case that Ernie is responsible whereas Sally is not.

8 By “full” responsibility we do not mean “as responsible as one can be.” Rather, we mean something along the lines of “the level of responsibility enjoyed by a typical responsible agent”.

9 Some may think that responsibility is zero-sum such that in such cases it transfers, in some sense, from the manipulee to the manipulator, or that the manipulee is responsible for the actions only to the extent that the manipulator is not. We do not think that a manipulator-focused view needs to be committed to this, nor do we interpret them as such. In order to block the concern that our intuition might be affected by something like this, the manipulator is sometimes stipulated to fail to meet standard conditions on responsibility (Mele 2006: 198, n. 16). For some arguments against such a transference view, or the claim that responsibility is, in some way, zero-sum, see Kaiserman (2021) and Sartorio (2016: 167-170).

10 Thanks to two anonymous referees for encouraging us to make these points explicit here.

11 Though see Yaffe’s discussion of the robotic tutor for the suggestion that the view *can* account for a particular type of case that may lack a manipulator (2003: 346). In discussing this case, Yaffe makes explicit that, despite the fact that the relevant “tracking” is often accompanied by an intention, it need not be.

12 Though Barnes (see Sect. 3) provides further criticism of Yaffe’s view (2015: 562–5), it’s not clear to us how Barnes’s view avoids the same issues (compare, for example, Barnes’s discussion of Yaffe’s view on p. 565, and the discussion of Barnes’s view on p. 575, n.22).

13 This last criterion is crucial, and exemplified in Yaffe’s discussion of “weakling” manipulators (2003: 346-8). In relation to the case of the Kaspar Hauser-type person, Yaffe offers a variation on which the monarch who imprisons him does not undermine his freedom: “…the monarch might be determined to prevent rescue at all costs and simply lack the control over his servants needed to see to it that attempted rescues would be prevented” (2003: 346).

14 Though Usher has a somewhat different, and highly intricate, view, his discussion suggests that it is the fact that manipulators are online that explains why some manipulated agents lack (full) responsibility. For more discussion of Usher’s view, see n. 25 below.

15 Despite Yaffe’s main focus on the facts that the manipulator tracks the victim and stands ready to reliably intervene, it is plausible to read him as having some further condition of this sort. All of the cases he considers do involve a manipulator actually intervening.

16 Waller considers similar cases (2014: 214, 221, n. 12).

17 Waller does consider something along these lines, though we do not find her response convincing. Waller’s way of distinguishing between Ernie and Carl is to suggest that, whereas the intention of Carl’s manipulator encompasses one action, “Diana’s intention casts a much wider scope—the way in which Diana designs Ernie’s zygote so that he *A*-s in 30 years dictates all of his actions. Thus, Diana’s intentional action of so constituting Ernie at his creation affects Ernie’s agency in a profound way and so has responsible” (2014: 217). We do not find this convincing. First, it is not clear how, according to *T**, this means that Ernie is substantially less responsible for *A*-ing, as opposed to just somewhat less responsible with respect to *other* actions and omissions. To be fair, this might result in reducing Ernie’s *derivative* responsibility for A-ing, insofar as it might reduce responsibility for actions from which this responsibility is partly derived from. Second, this wide scope of Diana’s intention is not clearly essential for the case, and we can get a similar case in which she merely intends for him to *A*, and has no pro- or con-attitude concerning any of his other actions.

18 As a reviewer helpfully points out, one might think that Carl, in the original case we present, truly is less deserving of blame for deciding to eat the snack (and for eating it). Thus, one might think that Waller’s view gives us the correct result, and our criticism fails. Yet, even if we grant that this is the right result, we suspect many will find a similar result implausible once we expand this to more ordinary interactions, like when Carl eats the snack on the basis of a suggestion from a friend (as opposed to a surreptitiously induced urge). But even if one *also* thinks that Carl is less deserving of blame when he acts on the basis of a friend’s suggestion, the main point remains: Waller’s view does not have the resources to distinguish between this case of Carl and the case of Ernie. This point, we think, is made clearer by slightly modifying the case of Ernie such that both Ernie and Bernie also act on a suggestion from a friend (which Diana foresaw in the case of Ernie). Both of these agents meet 1–3 of *T*.* We mention a possible solution below, in Sect. 6.1.

19 More recently, Gingerich makes a case for the value of what he calls *spontaneous freedom*, which is related to Barnes’s notion of creativity (2022). But since Gingerich takes such freedom to be independent of the freedom required for responsibility (p. 40), we set aside the view here.

20 Though this is a condition on an agent’s being *fully* free, some of Barnes’s discussion suggests a stronger view, on which he offers a necessary condition on an agent’s being free at all (see the introduction of PCC below). We read him as offering the weaker condition on full freedom.

21 Discussing Ernie and Bernie, Barnes explains that “[a] nearby possible world is one which differs from the actual world only in certain limited and contextually specified respects. In this case, the nearby possible worlds are those which resemble the actual world but in which there are differences which could bear on whether Ernie/Bernie perform the murder” (2015: 562). It is not clear to us how we are to understand this; whether Diana intends to modify Ernie’s zygote or not “could bear on whether [Ernie] perform[s] the murder,” it seems. Thus, on a plain reading, there is a nearby world in which Diana does not intend to modify his zygote, does not communicate anything to Ernie, and thus he acts creatively.

22 This is especially unclear since Barnes states that “This definition is meant to include ordinary types of communication (such as speech acts) but exclude acts by which an agent intentionally induces another to have a particular idea without intuitively communicating, such as A’s directing B’s attention to a sign containing information (a sign that A did not construct), or A’s giving B a few hints and waiting for B to grasp the idea by herself” (2015, 568). We are not sure how the definition could exclude cases like directing B’s attention or dropping hints without also excluding cases like Diana’s modification of Ernie’s zygote.

23 This may be along the lines of what Ismael is suggesting in some comments (2016: 104).

24 Herdova does not commit to the claims that (1) (a–f) are a complete list of elements of toolhood, (2) any of (a–f) is necessary for A’s being a tool of B, and (3) any of (a–f) alone is sufficient for A’s being a tool of B (2021: 258–9, 262 n. 3).

25 Usher’s view faces further issues not faced by DN’s. One issue concerns the fact that, since it appeals to the presence of an *online* manipulator, it faces the problem of dead manipulators we introduced when discussing Yaffe’s view. Second, and for this reason, Usher (2020: 320) claims that his view does not account for Ernie’s lack of responsibility (though see (Tierney and Glick 2020: 958, n. 6)). If this is right, then the view incurs the original cost of manipulator-focused views—failing to accommodate our intuitions in standard cases of natural forces—without gaining their advantage—accommodating our intuitions about agents in cases of original design.

26 Tierney, and Tierney and Glick, offer nuanced criticisms of these sorts of views, focusing on DN’s view (Tierney 2023; Tierney and Glick 2020). Put briefly, Tierney and Glick’s argument begins by showing that reliability (described in DN’s 1, introduced below) and stability (described in DN’s 2, also introduced below) can come apart, and this poses issues for a view like DN’s. We think Tierney and Glick’s arguments pose a significant challenge. Yet, we take a different route and do not elaborate on their arguments here. We do, however, explain that their discussion gives rise to a further objection to DN’s view (see n. 36 below).

27 For instance, they suggest that interventionism can be fruitfully applied to Frankfurt agents (2017: 1266–7, n. 11; 1274, n. 18), and that it can help to “explain away features of our experience of free agency that appear to implicate indeterminism” (2017: 1274, n. 18).

28 We follow DN as using CAS to refer to “features of the agent’s psychology that compatibilists typically judge as jointly (and minimally) sufficient for free will and moral responsibility” (2017: 1258).

29 “Seeing” if the value of *Y* changes involves plugging in the value for *X* into a formula that is part of the causal model, which yields a resulting value for *Y*.

30 We are very bad at darts.

31 Though, they also agree that “agents who are intentionally manipulated in the way that [Ernie] is lack free will and moral responsibility, since such agents are not the causal sources of their actions” (2017: 1267).

32 For a more developed discussion of different interpretations of DN’s view, see (Tierney and Glick 2020: 965–7).

33 From our reading, DN do not provide an argument for the claim that this relation is more *reliable*. Along with Tierney and Glick, we are not convinced that it is (2020: 962).

34 In a newer paper (2023), DN argue that for the manipulation argument to succeed, it is crucial that the manipulator have powers of perfect prediction. According to DN, in order to be an effective deterministic manipulator, one would need to have powers of perfect prediction, and if the manipulator is not a perfect predictor, then they cannot even form a relevant (effective) intention (2023: 463). We disagree. Humans are not perfect predictors, even though they commonly have (effective) intentions, including long-term ones. And one can exercise control over another, and manipulate them, without perfectly predicting how the other will behave. Perfect prediction is too high a bar for a manipulator to have control over their victim. DN also appeal to some empirical studies in support of this. We briefly discuss these below (see n. 38).

35 How might they handle such cases? Suppose that Joe and Rachel are unaware that using hot water in the kitchen redirects hot water flowing to the bathroom. If Joe uses hot water to wash the dishes while Rachel showers, his washing the dishes may be the casual source of Rachel’s cold shower. Similarly, in accidental results cases, the manipulators’ decision, intention, or intervention may be the causal source of the accidental results.

36 There is one potential issue worth mentioning, which, though they do not make it explicitly, follows from Tierney and Glick’s discussion (2020). In explaining stability and reliability of causal relations, Tierney and Glick offer a case of a mob boss ordering a hitman to kill a target (959-61). They argue that the boss’s decision is more stably related to the murder than is the hitman’s decision about how to do so. Yet, since it is up to the hitman to decide how and when the murder occurs, the hitman’s decision is more reliably related to the murder than is the mob boss’s; changes in the hitman’s decision more reliably predict changes in the murder. Because of this, neither relation is stronger than the other, on DN’s view. Assuming Tierney and Glick are right on this point, this implies that DN’s view faces difficulty with most cases mentioned above, including the standard one of Sweet Sally. This is because the manipulators in such cases can be seen as simply intending for Sally to kill the neighbor—and more like the mob boss—whereas Sally is more like the hitman who decides the time and means of the murder.

37 When introducing these cases, we offered another in which the manipulators intended for Sally to bake a pie for her neighbor, yet ended up with the same results of Sally committing murder. This reasons-based response, we suspect, will not be available for this sort of case.

38 For similar cases, see (Deery and Nahmias 2017: 1273; Pereboom and McKenna 2022: 192–3; Tierney 2023: 448, n. 8; Tierney and Glick 2020: 958–9, n. 7). DN briefly consider a case involving luck and accept what we take to be the counterintuitive result. Part of their reply relies on two studies which, they think, show common intuitions to align with their view. Thus, the intuitive pull of such cases is reduced (2017: 1271, n. 14) or, more strongly, “people are *far less* likely to have the intuition” if the manipulator is like a physical event (2023: 462, emphasis ours). We disagree with their interpretation of the studies, for reasons we can only briefly sketch. With respect to Phillips and Shaw’s work (2015), their studies show a small difference in blame judgements between cases in which the manipulee(s) perform the intended action, as opposed to when it is an accidental result, and these cases do not involve the sort of radical reversals that feature in our cases. Murray and Lombrozo (2017) have a sophisticated series of studies and analyses intended to probe *why* we intuit that subjects of standard manipulation are not (fully) responsible. Yet we do not think they provide strong support for DN’s conclusion. The relevant cases—one with a manipulator’s effective intention, and the other with natural forces—are different in more than one (potentially) relevant respect. The case including a manipulator’s effective intention involves a, presumably normal, businessman being drugged so that he “desperately want[s] to kill someone” and does so (2017: 453). This is contrasted with a case in which the businessman kills the person in front of him because he is the sort of person who would “rather kill someone than be scalded” (2017: 454), and does so after a natural force—a nut falling in his coffee—results in his flipping the table over and killing the person in front of him. In these two cases, not only is there a difference in terms of the presence of an effective manipulator, there is also, we think, a big difference in the significance of the change on the manipulee. Thus, there are (at least) two potential factors that could explain a difference in judgments of responsibility between the two cases, and we do not think they significantly blunt the force of cases involving lucky manipulators, accidental results, or natural forces.

39 The case of Fledgling Neurosurgeon is relevantly similar to Yaffe’s case of the weakling monarch (see n. 13 above).

40 For discussion, see (R. R. Waller 2014: 215, n. 6).

41 Having thought about whether Mac’s decision or intention is a casual source, we can also raise a potential worry for DN’s discussion of Frankfurt cases. DN suggest that the would-be intervener’s “deciding to do nothing is not an actual cause of [Frank’s choosing as he does]. So it is not the causal source of [his choice]” (2017: 1266, n. 11). But one might ask, instead, about the would-be intervener’s decision to ensure that Frank chooses as he does, and the conditional intention to intervene if necessary. The relation between this decision (or intention) and Frank’s choosing as he does, we think, would seem to exhibit relative stability and reliability. And recall that, on DN’s view, “X causes Y just in case, for at least some state of the model, there is an intervention on X that would reliably change the value of Y” (2017: 1261). Thus, it is not clear how, on this view, the would-be intervener’s decision to ensure (or resulting intention), is not the casual source of Frank’s choosing as he does. Perhaps of interest is a further variation on which Mac does not hit a rumble strip, but a second person, Tom, who has a machine just like Mac’s does, and thus accidentally modifies Claudia’s values in the same way. When Mac reaches Claudia’s house, he determines that he does not have to do anything and sits back while Claudia kills Pablo. If DN’s treatment of Frankfurt cases succeeds, then, Mac’s decision is not the causal source of Claudia’s action. But nor is the causal source an event concerning Tom, since those are not related to Claudia’s killing in any way that is more stable or robust than events concerning Mac in *Random Machine Induction*. We find it implausible that Claudia’s responsibility for the action hinges on whether it was Mac or Tom that hit this rumble strip.

42 One might think there is a further simple fix: require that the manipulator intentionally intervened. This would mean the view cannot account for Claudia’s lack of (full) responsibility in Lucky Machine Induction, but it would also mean the view is consistent across the two cases of machine induction. We do think, however, that such a revision would be in tension with other parts of Yaffe’s view. As he suggests when discussing the Robotic Tutor (2003: 346-8), the “manipulator” need not even have an intention (or the capacity to form one) in order to track the pattern of response to reasons in the right way. It would seem strange, we think, for such a “manipulator” to intervene intentionally.

43 For the sort of cases we have in mind, see (Cyr 2016, 2020a; Haji and Cuypers 2001; King 2013; McKenna 2016; Mele 2019: 124–6).

44 Notice, however, that if the manipulators instead intended for Sally to *kill either George or his son*, or *kill someone from George’s household*, these views would then be able to account for the intuition that Sally lacks (full) responsibility in either case.

45 It is not always clear what, precisely, Yaffe has in mind in relation to this; i.e., it is not clear what actions, in particular, a manipulee lacks (full) responsibility for. For instance, is Sally (fully) responsible for obnoxiously honking her horn (as she normally does) on her way to buy a gun for killing George?

46 Skill, we suspect, will correlate well with equifinality (Deery and Nahmias 2017: 1263–4).

47 We do not consider cases of mild manipulation, like Carl’s, with respect to interventionist views, since this could be a paper of its own. Roughly put, how such views apply will depend significantly on further features of the case. For instance, on whether by implanting a mild desire, the manipulator was doing the most that they were capable of, or just the least they needed, while capable of doing much more.

48 For discussion of interventionist views and Frankfurt cases, see n. 41.

49 For some discussion, see (Herdova 2021: 261, 262 n. 3).

50 For further issues that different bypassing views face, see (Cyr 2020b; De Marco 2022: 1960–1, 2023a: 1440–43).

51 Of course, as we have already noted (e.g., in discussing the Ernie case), some cases favor manipulator-focused views over bypassing views, too (see also n. 50 above).
